# Difference between age-related macular degeneration and polypoidal choroidal vasculopathy in the hereditary contribution of the A69S variant of the age-related maculopathy susceptibility 2 gene (*ARMS2*)

**Published:** 2011-12-31

**Authors:** Suiho Yanagisawa, Naoshi Kondo, Akiko Miki, Wataru Matsumiya, Sentaro Kusuhara, Yasutomo Tsukahara, Shigeru Honda, Akira Negi

**Affiliations:** Department of Surgery, Division of Ophthalmology, Kobe University Graduate School of Medicine, Chuo-ku, Kobe, Japan

## Abstract

**Purpose:**

To investigate whether the A69S variant of the age-related maculopathy susceptibility 2 gene (*ARMS2*) has a different hereditary contribution in neovascular age-related macular degeneration (AMD) and polypoidal choroidal vasculopathy (PCV).

**Methods:**

We initially conducted a comparative genetic analysis of neovascular AMD and PCV, genotyping the *ARMS2* A69S variant in 181 subjects with neovascular AMD, 198 subjects with PCV, and 203 controls in a Japanese population. Genotyping was conducted using TaqMan technology. Results were then integrated into a meta-analysis of previous studies representing an assessment of the association between the *ARMS2* A69S variant and neovascular AMD and/or PCV, comprising a total of 3,828 subjects of Asian descent. The Q-statistic test was used to assess between-study heterogeneity. Summary odds ratios (ORs) and 95% confidence intervals (CIs) were estimated using a fixed effects model.

**Results:**

The genetic effect of the A69S variant was stronger in neovascular AMD (allelic summary OR=3.09 [95% CI, 2.71–3.51], fixed effects p<0.001) than in PCV (allelic summary OR=2.13 [95% CI, 1.91–2.38], fixed effects p<0.001). The pooled risk allele frequency was significantly higher in neovascular AMD (64.7%) than in PCV (55.6%). The population attributable risks for the variant allele were estimated to be 43.9% (95% CI, 39.0%–48.4%) and 29.7% (95% CI, 25.4%–34.0%) for neovascular AMD and PCV, respectively. No significant between-study heterogeneity was observed in any statistical analysis in this meta-analysis.

**Conclusions:**

Our meta-analysis provides substantial evidence that the *ARMS2* A69S variant confers a significantly higher risk of neovascular AMD than PCV. Furthermore, there is compelling evidence that the risk attributable to the A69S variant differs between geographic atrophy and neovascular AMD. Together with defining the molecular basis of susceptibility, understanding the relationships between this genomic region and disease subtypes will yield important insights, elucidating the biologic architecture of this phenotypically heterogeneous disorder.

## Introduction

Age-related macular degeneration (AMD), a leading cause of irreversible blindness among older individuals in developed countries, is a common multifactorial disease with heterogeneous clinical manifestations [1]. An early hallmark lesion of AMD is large drusen and pigmentary abnormalities in the retinal pigment epithelium of the macula. The advanced form of the disease is classified into two main groups: “dry” and “wet” types; the former is characterized by geographic atrophy and the latter by the development of choroidal neovascularization (CNV) in the central macula (neovascular AMD). AMD has divergent clinical features between racial groups, and the ratio of neovascular AMD to dry AMD is higher in Asian than in European populations [2-5].

Remarkable progress has recently been made in understanding the genetic basis of AMD. Several AMD susceptibility loci have been established with convincing statistical evidence, including the complement factor H gene on chromosome 1q32 [6-8], two tightly linked genes on 10q26 (age-related maculopathy susceptibility 2 [*ARMS2*] [9] and high-temperature requirement factor H [*HTRA1*]) [10,11], the complement component 3 gene on 19p13 [12], two neighboring genes on 6p21 (complement factor B and complement component 2) [13], the complement factor I gene on 4q25 [14], the hepatic lipase gene on 15q22 [15,16], the cholesterylester transfer protein gene on 16q21 [15,16], and the tissue inhibitor of the metalloproteinase 3 gene on 22q12 [15,16]. Recent additions to the growing list of potential AMD risk loci include 6q21–q22.3 that encompass two genes—the collagen, type X, alpha 1 gene and the fyn-related kinase gene—and 6p12 harboring the vascular endothelial growth factor A gene, which were identified through a recent large-scale meta-analysis of genome-wide association study for advanced AMD [17]. The meta-analysis showed that the missense allele encoding A69S (rs10490924) in *ARMS2* confers the strongest disease risk, among others [17].

Polypoidal choroidal vasculopathy (PCV), characterized by inner choroidal vascular networks ending in polypoidal lesions [18], is now clinically classified as a specific type of AMD [19]. PCV is particularly prevalent in Asian populations, accounting for 54.7% of patients with the neovascular form of AMD in the Japanese population [20] and 24.5% in the Chinese population [21], but only 8% to 13% in Caucasians [22]. PCV shares many similarities with neovascular AMD, including demography [20], pathology [23,24], and manifestation [20]; however, important differences have been noted in histopathology [25], clinical behavior [22], and response to therapy [18,26]. These similarities and differences have been a subject of much interest and debate regarding whether the vascular abnormality in PCV represents neovascularization or a phenotype distinct from CNV [23-25,27].

We have previously shown that the *ARMS2* A69S variant is strongly associated with neovascular AMD and PCV, with a stronger association in neovascular AMD than in PCV [28]; however, the difference was not statistically significant, probably owing to a limitation in statistical power. Subsequent A69S association studies have consistently reported a trend toward stronger evidence for association in neovascular AMD than in PCV [29-31]. Interestingly, a significant difference in genetic susceptibility between geographic atrophy and neovascular AMD has been repeatedly observed at this locus [17,32]. Sub-phenotype associations are currently being actively researched in complex diseases, such as inflammatory bowel disease [33], rheumatoid arthritis [34], and various cancers [35-37]. Genotype–phenotype correlations between risk alleles and disease subtypes may provide an insight into the underlying etiologic pathways of complex diseases.

To date, some meta-analyses have been published regarding the association between AMD and the *ARMS2*/*HTRA1* region [38-40], but none of these studies focused on PCV. Here we conducted a comparative genetic analysis of neovascular AMD and PCV in our original sample set of Japanese ancestry, genotyping the *ARMS2* A69S variant in 181 subjects with neovascular AMD, 198 subjects with PCV, and 203 controls. Results were then integrated into a meta-analysis of previous studies representing an assessment of the association between the *ARMS2* A69S variant and neovascular AMD and/or PCV, comprising a total of 3,828 subjects of Asian descent, to more reliably compare the genetic effect of *ARMS2* A69S between neovascular AMD and PCV.

## Methods

### New data set: Study participants

The study protocol was approved by the Institutional Review Board at Kobe University Graduate School of Medicine and performed in accordance with the Declaration of Helsinki. Written informed consent was obtained from all subjects before participation in this study. All cases and controls included in our original sample set were Japanese individuals recruited from the Department of Ophthalmology at Kobe University Hospital in Kobe, Japan. This cohort is an extension of one previously published for an association with the *ARMS2* A69S variant [28]. A portion of the subjects in the present study had participated in our previous studies in which phenotyping criteria were fully described [28,41,42]. In brief, all our subjects with neovascular AMD and PCV underwent a comprehensive ophthalmic examination including indocyanine green angiography, and were defined as having angiographically well defined lesions of CNV or PCV. The controls were not related to the cases and were defined as individuals without macular degeneration and changes such as drusen or pigment abnormalities, and were thus categorized as having clinical age-related maculopathy staging system stage 1 [43]. The demographic details of the study subjects are listed in Table 1.

**Table 1:** Characteristics of the Study Population

**Groups**	**Neovascular AMD**	**PCV**	**Control**
Number of subjects	181	198	203
Gender (male/female)	139/42	157/41	120/83
Mean age ± SD (years)	75±7.4	73±7.3	72±6.0
Age range (years)	55–94	54–93	56–95

Abbreviations: AMD, age-related macular degeneration; PCV, polypoidal choroidal vasculopathy; SD, standard deviation.

### Genotyping

Genomic DNA was extracted from peripheral blood using a standard methodology. Genotyping was performed using a pre-developed TaqMan SNP Genotyping Assay (Assay ID: C_29934973_20; Applied Biosystems, Foster City, CA) on a StepOnePlus™ Real-Time PCR System (Applied Biosystems) in accordance with the manufacturer’s recommendations.

### Statistical analysis

Allelic associations were evaluated for the *ARMS2* A69S variant with chi-square tests on 2 × 2 contingency tables using the software package PLINK v1.07. Deviations from the Hardy–Weinberg equilibrium (HWE) were tested using the exact test [44] implemented in PLINK. The odds ratio (OR) and corresponding 95% confidence interval (CI) were calculated relative to the major allele. Genotype-specific ORs were estimated for the heterozygous (GT) and risk homozygous (TT) genotypes, with the common homozygous (GG) genotype the baseline category with unconditional logistic regression using the JMP software (version 6.0.3; SAS Institute, Cary, NC). To test for heterogeneity between ORs for neovascular AMD and PCV, we conducted a logistic regression analysis of the cases (case-only analysis) using R project, where the subtypes were used as the outcome and the A69S genotype as the explanatory variable [45].

### Meta-analysis: Identification and eligibility of relevant studies

We performed a systematic PubMed literature search (up to May 2011) using the following search terms in different combinations: “HtrA serine peptidase 1” or “*HTRA1*,” “age-related maculopathy susceptibility 2,” “*ARMS2,*” or “LOC387715,” and “age-related macular degeneration” or “polypoidal choroidal vasculopathy.” The literature search was performed in duplicate by two authors (S.Y. and N.K.).

Studies included in the meta-analysis had to fulfill the following criteria: (1) The study must be unrelated case-control or population-based representing an assessment of the association between the *ARMS2* A69S variant and neovascular AMD and/or PCV in East Asian populations. (2) The study must distinguish PCV from the neovascular form of AMD based on findings of indocyanine green angiography, and must look at PCV and/or neovascular AMD (CNV) as specific outcomes. (3) The study must present available data on allele and genotype distributions for cases and controls. (4) The study must be written in English and published in peer-reviewed journals. For duplicate publications, the largest data set was chosen for meta-analysis.

### Data extraction

The following variables were extracted from each study: the name of the first author, the year of publication, ethnicity, and allele and genotype distributions in cases and controls.

### Statistical analyses

For each study, deviations from the HWE in controls were tested using the exact test [44]. Pooled allele and genotype frequencies of the A69S variant were estimated with the fixed effects model [46] if heterogeneity among studies was absent, or with the random effects model [47] if heterogeneity was present. We estimated summary ORs and 95% CIs according to the Mantel–Haenszel fixed effects model [46] if heterogeneity among studies was absent or the DerSimonian–Laird random effects model [47] if there was evidence of between-study heterogeneity. The population attribute risk was calculated to demonstrate the number of cases in the total population that could be attributed to the risk genotype, as described previously [48].

Between-study heterogeneity was assessed using the Q-statistic test and *I*^2^ statistic [49,50]. A p value of <0.1 was considered statistically significant for the Q-statistic test. *I*^2^ ranges between 0% and 100% (where a value of 0% represents no heterogeneity), and larger values represent increasing heterogeneity.

All meta-analyses were conducted using the Stata software (version 11.0; Stata Corporation, College Station, TX). All tests were two tailed. A p value of <0.05 was considered statistically significant except for the test of between-study heterogeneity.

## Results

### Comparative genetic analysis in our original sample set

We initially conducted a comparative genetic analysis of neovascular AMD and PCV, genotyping the *ARMS2* A69S variant (rs10490924) in our original sample set. Genotype distributions for this variant are given in Table 2, along with those of other studies included in the subsequent meta-analysis. No departure from the HWE was observed at this variant among the controls (p=0.88). As expected, the *ARMS2* A69S variant showed strong evidence of association with neovascular AMD and PCV. ORs for the risk allele T were 2.82 (95% CI, 2.10–3.78, p=2.4×10^−12^) and 2.39 (95% CI, 1.80–3.17, p=1.3×10^−9^) for neovascular AMD and PCV, respectively. For heterozygous and homozygous carriers of the risk allele, the genotype-specific OR was 2.62 (95% CI, 1.55–4.52) and 7.49 (95% CI, 4.11–14.07) for neovascular AMD and 1.56 (95% CI, 0.97–2.53) and 5.02 (95% CI, 2.89–8.90) for PCV, respectively. Similar to previous findings of *ARMS2* A69S association studies [29-31], the variant showed a trend toward stronger effect in neovascular AMD than in PCV. However, a case-only heterogeneity test with logistic regression analysis showed a nonsignificant value in our original sample set (heterogeneity p=0.31), possibly reflecting inadequate statistical power in this single study. Our own data were then combined with those from previously published studies in the subsequent meta-analysis.

**Table 2:** Allele and Genotype Distributions of the *ARMS2* A69S Variant of Case-Control Studies Contributing to the Meta-Analysis

			**Genotype (GG/GT/TT)**			**Risk allele frequency**			
**Study**	**Year**	**Ethnicity**	**Neovascular AMD**	**PCV**	**Control**	**Neovascular AMD**	**PCV**	**Control**	PHWE*
[51]	2008	Japanese	NA	15/49/45	39/32/14	NA	0.64	0.35	0.10
[52]	2008	Chinese	NA	17/30/25	33/48/12	NA	0.56	0.39	0.51
[29]	2009	Japanese	18/30/52	18/50/32	85/84/20	0.67	0.57	0.33	1.0
[30]	2010	Japanese	67/155/183	122/216/171	502/638/196	0.64	0.55	0.39	0.82
[31]	2011	Japanese	6/20/24	22/20/18	64/58/16	0.68	0.47	0.33	0.70
This study	2011	Japanese	26/81/74	42/77/79	79/94/30	0.63	0.59	0.38	0.88

Abbreviations: ARMS2, age-related maculopathy susceptibility 2; AMD, age-related macular degeneration; PCV, polypoidal choroidal vasculopathy; HWE, Hardy–Weinberg equilibrium; NA, not available. *p values generated by the exact test for Hardy–Weinberg equilibrium.

### Meta-analysis: Eligibility of studies

Our search identified five studies that met our inclusion criteria [29-31,51,52]. Data from these five studies and our original study were combined for the meta-analysis. Table 2 lists the studies included in the meta-analysis. The combined sample size for this meta-analysis was 3,828.

### Allele and genotype frequency

None of the five previously published studies demonstrated significant deviation from the HWE among controls (Table 2). To estimate the pooled frequency of the A69S variant in Asian populations, we used allele data from controls. The pooled frequency for the risk allele T was 37.4% (95% CI, 35.9–38.8), and individuals carrying at least one copy of the risk allele (GT + TT) accounted for 60.8% (95% CI, 58.7–62.9) of the control populations. No evidence of heterogeneity in these frequencies was observed among controls across the six studies (allele frequency, Q=8.50, 5 degrees of freedom [d.f.], p=0.13, *I*^2^=41.2%; frequency of GT/TT genotypes, Q=9.0, 5 d.f., p=0.11, *I*^2^=44.5%).

### Quantitative synthesis

**Figure 1:**
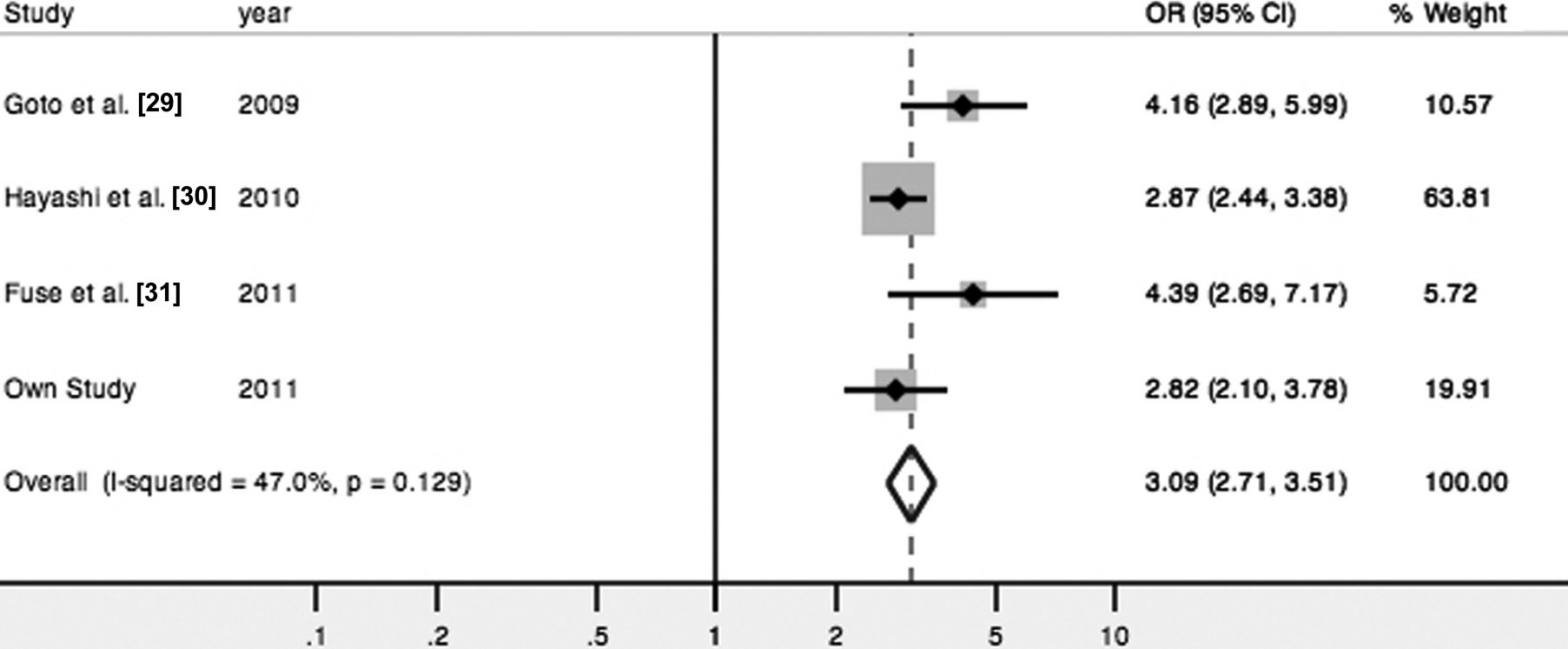
Forest plot showing the association between *ARMS2* A69S and neovascular age-related macular degeneration. Odds ratios (black squares) and 95% confidence intervals (bars) are given for each study. Also shown are the unshaded diamonds of the summary odds ratio based on the Mantel–Haenszel fixed effects model.

**Figure 2:**
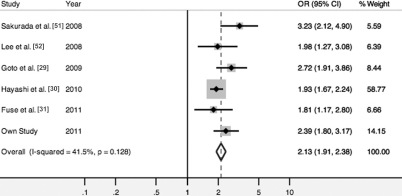
Forest plot showing the association between *ARMS2* A69S and polypoidal choroidal vasculopathy. Odds ratios (black squares) and 95% confidence intervals (bars) are given for each study. Also shown are the unshaded diamonds of the summary odds ratio based on the Mantel–Haenszel fixed effects model.

We conducted a meta-analysis based on an allele contrast model. The A69S variant showed a significant summary OR of 3.09 ([95% CI, 2.71–3.51], fixed effects p<0.001; Figure 1) for neovascular AMD and 2.13 ([95% CI, 1.91–2.38], fixed effects p<0.001; Figure 2) for PCV. The Q-statistic test showed no significant between-study heterogeneity in association tests for neovascular AMD or PCV (p>0.1; Figure 1 and Figure 2). The population attribute risks for the risk allele were 43.9% (95% CI, 39.0%–48.4%) and 29.7% (95% CI, 25.4%–34.0%) for neovascular AMD and PCV, respectively. Next, we compared the allele frequencies of the variant between the two subtypes, combining data from four studies that included neovascular AMD and PCV subtypes in the case groups (Table 2). The pooled risk allele frequency was significantly higher in neovascular AMD than in PCV (64.7% versus 55.6%; p<0.001), without heterogeneity across studies (Q=5.38, 3 days.f., p=0.15, *I*^2^=44.3%). This result, coupled with the finding that the 95% CIs for allelic summary ORs for neovascular AMD did not overlap with those for PCV, indicates that the genetic effect of the *ARMS2* A69S variant is significantly stronger in neovascular AMD than in PCV.

## Discussion

Several studies have reported that the *ARMS2* A69S variant is strongly associated with neovascular AMD and PCV, with a stronger association in neovascular AMD than in PCV [29-31]. However, the differences between the two were not statistically significant in most studies, probably owing to a limitation in the statistical power. Our meta-analysis has revealed that the *ARMS2* A69S variant confers a significantly greater risk of neovascular AMD than of PCV. The pooled risk allele frequency was significantly higher in neovascular AMD (64.7%) compared with PCV (55.6%). The meta-analysis estimated the attributable risks for the variant allele were 43.9% and 29.7% for neovascular AMD and PCV, respectively. In the control populations, the pooled frequency for the risk allele T of the A69S variant was estimated to be 37.4%, and individuals carrying at least one copy of the risk allele accounted for 60.8%, indicating its population-wide epidemiological consequence in Asian populations owing to the high frequency of the risk allele. No significant between-study heterogeneity was observed in any statistical analysis in this meta-analysis of Asian populations.

There is increasing evidence that ethnicity influences disease via genetic background [53]. Risk allele frequencies of A69S diverge greatly between European and Asian populations from the HapMap sample, with almost 40% risk allele frequencies in Asian populations compared to 20% in individuals of European descent. The distributions of the neovascular subtype of AMD differ markedly between European and Asian populations, and parallel the risk allele frequencies of this variant, with Asians having a much higher rate of the neovascular subtype than Europeans [2-5], suggesting that this locus may contribute to ethnic heterogeneity in the manifestation of AMD subtypes.

Currently, how the *ARMS2*/*HTRA1* region on 10q26 is a source of genetic risk for AMD is unclear. Much effort has been made to localize variant(s) causally related to AMD in this region and to understand the molecular basis of the susceptibility [10,11,54-58]. However, there is high linkage disequilibrium (LD) across the *ARMS2*/*HTRA1* region, adding to the difficulty in identifying true causal variant(s) by association mapping alone [55]. The association signal at 10q26 converges on a region of an extensive LD block spanning *ARMS2* and *HTRA1* [54,55]. This LD block harbors multiple susceptibility alleles of which the *ARMS2* A69S variant has been reported to show the strongest evidence for association [54]. Two variants within this LD block that were correlated with A69S through strong LD—SNP rs11200638 in the promoter of *HTRA1* [10,11] and the insertion/deletion polymorphism (c.(*)372_815del443ins54) in the 3′-UTR region of *ARMS2* [55]—have recently been proposed as causal variants based on mechanistic functional evidence, but there is no agreement across studies [10,11,54-58]. Thus, the molecular basis of the susceptibility remains obscure.

In conclusion, our meta-analysis has identified a difference in the hereditary contribution of the *ARMS2* A69S variant between neovascular AMD and PCV. In addition, a significant difference has been reported between geographic atrophy and neovascular AMD with respect to genetic susceptibility at this locus [17,32]. This fact, coupled with our findings, indicates that the risk attributable to the A69S variant differs among AMD subtypes. Given the importance of the *ARMS2*/*HTRA1* region on 10q26 in AMD susceptibility, defining molecular mechanisms through which the genomic variants influence disease risk and understanding the relationships between this region and disease subtypes will yield important insights, elucidating the biologic architecture of this phenotypically heterogeneous disorder.

## References

[1] Jager RD, Mieler WF, Miller JW (2008). Age-related macular degeneration.. N Engl J Med.

[2] Klein R, Klein BE, Linton KL (1992). Prevalence of age-related maculopathy. The Beaver Dam Eye Study.. Ophthalmology.

[3] Mitchell P, Smith W, Attebo K, Wang JJ (1995). Prevalence of age-related maculopathy in Australia. The Blue Mountains Eye Study.. Ophthalmology.

[4] Oshima Y, Ishibashi T, Murata T, Tahara Y, Kiyohara Y, Kubota T (2001). Prevalence of age related maculopathy in a representative Japanese population: the Hisayama study.. Br J Ophthalmol.

[5] Kawasaki R, Yasuda M, Song SJ, Chen SJ, Jonas JB, Wang JJ, Mitchell P, Wong TY (2010). The prevalence of age-related macular degeneration in Asians: a systematic review and meta-analysis.. Ophthalmology.

[6] Klein RJ, Zeiss C, Chew EY, Tsai JY, Sackler RS, Haynes C, Henning AK, SanGiovanni JP, Mane SM, Mayne ST, Bracken MB, Ferris FL, Ott J, Barnstable C, Hoh J (2005). Complement factor H polymorphism in age-related macular degeneration.. Science.

[7] Edwards AO, Ritter R, Abel KJ, Manning A, Panhuysen C, Farrer LA (2005). Complement factor H polymorphism and age-related macular degeneration.. Science.

[8] Haines JL, Hauser MA, Schmidt S, Scott WK, Olson LM, Gallins P, Spencer KL, Kwan SY, Noureddine M, Gilbert JR, Schnetz-Boutaud N, Agarwal A, Postel EA, Pericak-Vance MA (2005). Complement factor H variant increases the risk of age-related macular degeneration.. Science.

[9] Rivera A, Fisher SA, Fritsche LG, Keilhauer CN, Lichtner P, Meitinger T, Weber BH (2005). Hypothetical LOC387715 is a second major susceptibility gene for age-related macular degeneration, contributing independently of complement factor H to disease risk.. Hum Mol Genet.

[10] Dewan A, Liu M, Hartman S, Zhang SS, Liu DT, Zhao C, Tam PO, Chan WM, Lam DS, Snyder M, Barnstable C, Pang CP, Hoh J (2006). *HTRA1* promoter polymorphism in wet age-related macular degeneration.. Science.

[11] Yang Z, Camp NJ, Sun H, Tong Z, Gibbs D, Cameron DJ, Chen H, Zhao Y, Pearson E, Li X, Chien J, Dewan A, Harmon J, Bernstein PS, Shridhar V, Zabriskie NA, Hoh J, Howes K, Zhang K (2006). A variant of the *HTRA1* gene increases susceptibility to age-related macular degeneration.. Science.

[12] Yates JR, Sepp T, Matharu BK, Khan JC, Thurlby DA, Shahid H, Clayton DG, Hayward C, Morgan J, Wright AF, Armbrecht AM, Dhillon B, Deary IJ, Redmond E, Bird AC, Moore AT, Genetic Factors in AMD Study Group (2007). Complement C3 variant and the risk of age-related macular degeneration.. N Engl J Med.

[13] Gold B, Merriam JE, Zernant J, Hancox LS, Taiber AJ, Gehrs K, Cramer K, Neel J, Bergeron J, Barile GR, Smith RT, Hageman GS, Dean M, Allikmets R, AMD Genetics Clinical Study Group (2006). Variation in factor B (*BF*) and complement component 2 (*C2*) genes is associated with age-related macular degeneration.. Nat Genet.

[14] Fagerness JA, Maller JB, Neale BM, Reynolds RC, Daly MJ, Seddon JM (2009). Variation near complement factor I is associated with risk of advanced AMD.. Eur J Hum Genet.

[15] Neale BM, Fagerness J, Reynolds R, Sobrin L, Parker M, Raychaudhuri S, Tan PL, Oh EC, Merriam JE, Souied E, Bernstein PS, Li B, Frederick JM, Zhang K, Brantley MA, Lee AY, Zack DJ, Campochiaro B, Campochiaro P, Ripke S, Smith RT, Barile GR, Katsanis N, Allikmets R, Daly MJ, Seddon JM (2010). Genome-wide association study of advanced age-related macular degeneration identifies a role of the hepatic lipase gene (LIPC).. Proc Natl Acad Sci USA.

[16] Chen W, Stambolian D, Edwards AO, Branham KE, Othman M, Jakobsdottir J, Tosakulwong N, Pericak-Vance MA, Campochiaro PA, Klein ML, Tan PL, Conley YP, Kanda A, Kopplin L, Li Y, Augustaitis KJ, Karoukis AJ, Scott WK, Agarwal A, Kovach JL, Schwartz SG, Postel EA, Brooks M, Baratz KH, Brown WL, Brucker AJ, Orlin A, Brown G, Ho A, Regillo C, Donoso L, Tian L, Kaderli B, Hadley D, Hagstrom SA, Peachey NS, Klein R, Klein BE, Gotoh N, Yamashiro K, Ferris Iii F, Fagerness JA, Reynolds R, Farrer LA, Kim IK, Miller JW, Cortón M, Carracedo A, Sanchez-Salorio M, Pugh EW, Doheny KF, Brion M, Deangelis MM, Weeks DE, Zack DJ, Chew EY, Heckenlively JR, Yoshimura N, Iyengar SK, Francis PJ, Katsanis N, Seddon JM, Haines JL, Gorin MB, Abecasis GR, Swaroop A, Complications of Age-Related Macular Degeneration Prevention Trial Research Group (2010). Genetic variants near TIMP3 and high-density lipoprotein-associated loci influence susceptibility to age-related macular degeneration.. Proc Natl Acad Sci USA.

[17] Yu Y, Bhangale TR, Fagerness J, Ripke S, Thorleifsson G, Tan PL, Souied EH, Richardson AJ, Merriam JE, Buitendijk GH, Reynolds R, Raychaudhuri S, Chin KA, Sobrin L, Evangelou E, Lee PH, Lee AY, Leveziel N, Zack DJ, Campochiaro B, Campochiaro P, Smith RT, Barile GR, Guymer RH, Hogg R, Chakravarthy U, Robman LD, Gustafsson O, Sigurdsson H, Ortmann W, Behrens TW, Stefansson K, Uitterlinden AG, van Duijn CM, Vingerling JR, Klaver CC, Allikmets R, Brantley MA, Baird PN, Katsanis N, Thorsteinsdottir U, Ioannidis JP, Daly MJ, Graham RR, Seddon JM (2011). Common Variants near FRK/COL10A1 and VEGFA are Associated with Advanced Age-related Macular Degeneration.. Hum Mol Genet.

[18] Imamura Y, Engelbert M, Iida T, Freund KB, Yannuzzi LA (2010). Polypoidal choroidal vasculopathy: a review.. Surv Ophthalmol.

[19] Takahashi K, Ishibashi T, Ogur Y, Yuzawa M, Working Group for Establishing Diagnostic Criteria for Age-Related Macular Degeneration. (2008). Classification and diagnostic criteria of age-related macular degeneration.. Nippon Ganka Gakkai Zasshi.

[20] Maruko I, Iida T, Saito M, Nagayama D, Saito K (2007). Clinical characteristics of exudative age-related macular degeneration in Japanese patients.. Am J Ophthalmol.

[21] Liu Y, Wen F, Huang S, Luo G, Yan H, Sun Z, Wu D (2007). Subtype lesions of neovascular age-related macular degeneration in Chinese patients.. Graefes Arch Clin Exp Ophthalmol.

[22] Ciardella AP, Donsoff IM, Huang SJ, Costa DL, Yannuzzi LA (2004). Polypoidal choroidal vasculopathy.. Surv Ophthalmol.

[23] Kikuchi M, Nakamura M, Ishikawa K, Suzuki T, Nishihara H, Yamakoshi T, Nishio K, Taki K, Niwa T, Hamajima N, Terasaki H (2007). Elevated C-reactive protein levels in patients with polypoidal choroidal vasculopathy and patients with neovascular age-related macular degeneration.. Ophthalmology.

[24] Tong JP, Chan WM, Liu DT, Lai TY, Choy KW, Pang CP, Lam DS (2006). Aqueous humor levels of vascular endothelial growth factor and pigment epithelium-derived factor in polypoidal choroidal vasculopathy and choroidal neovascularization.. Am J Ophthalmol.

[25] Nakashizuka H, Mitsumata M, Okisaka S, Shimada H, Kawamura A, Mori R, Yuzawa M (2008). Clinicopathologic findings in polypoidal choroidal vasculopathy.. Invest Ophthalmol Vis Sci.

[26] Honda S, Imai H, Yamashiro K, Kurimoto Y, Kanamori-Matsui N, Kagotani Y, Tamura Y, Yamamoto H, Ohoto S, Takagi H, Uenishi M, Negi A (2009). Comparative assessment of photodynamic therapy for typical age-related macular degeneration and polypoidal choroidal vasculopathy: a multicenter study in Hyogo prefecture, Japan.. Ophthalmologica.

[27] Yuzawa M, Mori R, Kawamura A (2005). The origins of polypoidal choroidal vasculopathy.. Br J Ophthalmol.

[28] Kondo N, Honda S, Ishibashi K, Tsukahara Y, Negi A (2007). *LOC387715*/*HTRA1* variants in polypoidal choroidal vasculopathy and age-related macular degeneration in a Japanese population.. Am J Ophthalmol.

[29] Goto A, Akahori M, Okamoto H, Minami M, Terauchi N, Haruhata Y, Obazawa M, Noda T, Honda M, Mizota A, Tanaka M, Hayashi T, Tanito M, Ogata N, Iwata T (2009). Genetic analysis of typical wet-type age-related macular degeneration and polypoidal choroidal vasculopathy in Japanese population.. J Ocul Biol Dis Infor.

[30] Hayashi H, Yamashiro K, Gotoh N, Nakanishi H, Nakata I, Tsujikawa A, Otani A, Saito M, Iida T, Matsuo K, Tajima K, Yamada R, Yoshimura N (2010). *CFH* and *ARMS2* variations in age-related macular degeneration, polypoidal choroidal vasculopathy, and retinal angiomatous proliferation.. Invest Ophthalmol Vis Sci.

[31] Fuse N, Mengkegale M, Miyazawa A, Abe T, Nakazawa T, Wakusawa R, Nishida K (2011). Polymorphisms in *ARMS2* (*LOC387715*) and *LOXL1* genes in the Japanese with age-related macular degeneration.. Am J Ophthalmol.

[32] Sobrin L, Reynolds R, Yu Y, Fagerness J, Leveziel N, Bernstein PS, Souied EH, Daly MJ, Seddon JM (2011). *ARMS2*/*HTRA1* locus can confer differential susceptibility to the advanced subtypes of age-related macular degeneration.. Am J Ophthalmol.

[33] Franke A, Balschun T, Karlsen TH, Sventoraityte J, Nikolaus S, Mayr G, Domingues FS, Albrecht M, Nothnagel M, Ellinghaus D, Sina C, Onnie CM, Weersma RK, Stokkers PC, Wijmenga C, Gazouli M, Strachan D, McArdle WL, Vermeire S, Rutgeerts P, Rosenstiel P, Krawczak M, Vatn MH, Mathew CG, Schreiber S, IBSEN Study Group (2008). Sequence variants in *IL10, ARPC2* and multiple other loci contribute to ulcerative colitis susceptibility.. Nat Genet.

[34] Thomson W, Barton A, Ke X, Eyre S, Hinks A, Bowes J, Donn R, Symmons D, Hider S, Bruce IN, Wilson AG, Marinou I, Morgan A, Emery P, Wellcome Trust Case Control Consortium (2007). YEAR Consortium; Carter A, Steer S, Hocking L, Reid DM, Wordsworth P, Harrison P, Strachan D, Worthington J. Rheumatoid arthritis association at 6q23.. Nat Genet.

[35] Stacey SN, Manolescu A, Sulem P, Thorlacius S, Gudjonsson SA, Jonsson GF, Jakobsdottir M, Bergthorsson JT, Gudmundsson J, Aben KK, Strobbe LJ, Swinkels DW, van Engelenburg KC, Henderson BE, Kolonel LN, Le Marchand L, Millastre E, Andres R, Saez B, Lambea J, Godino J, Polo E, Tres A, Picelli S, Rantala J, Margolin S, Jonsson T, Sigurdsson H, Jonsdottir T, Hrafnkelsson J, Johannsson J, Sveinsson T, Myrdal G, Grimsson HN, Sveinsdottir SG, Alexiusdottir K, Saemundsdottir J, Sigurdsson A, Kostic J, Gudmundsson L, Kristjansson K, Masson G, Fackenthal JD, Adebamowo C, Ogundiran T, Olopade OI, Haiman CA, Lindblom A, Mayordomo JI, Kiemeney LA, Gulcher JR, Rafnar T, Thorsteinsdottir U, Johannsson OT, Kong A, Stefansson K (2008). Common variants on chromosome 5p12 confer susceptibility to estrogen receptor-positive breast cancer.. Nat Genet.

[36] Goode EL, Chenevix-Trench G, Song H, Ramus SJ, Notaridou M, Lawrenson K, Widschwendter M, Vierkant RA, Larson MC, Kjaer SK, Birrer MJ, Berchuck A, Schildkraut J, Tomlinson I, Kiemeney LA, Cook LS, Gronwald J, Garcia-Closas M, Gore ME, Campbell I, Whittemore AS, Sutphen R, Phelan C, Anton-Culver H, Pearce CL, Lambrechts D, Rossing MA, Chang-Claude J, Moysich KB, Goodman MT, Dörk T, Nevanlinna H, Ness RB, Rafnar T, Hogdall C, Hogdall E, Fridley BL, Cunningham JM, Sieh W, McGuire V, Godwin AK, Cramer DW, Hernandez D, Levine D, Lu K, Iversen ES, Palmieri RT, Houlston R, van Altena AM, Aben KK, Massuger LF, Brooks-Wilson A, Kelemen LE, Le ND, Jakubowska A, Lubinski J, Medrek K, Stafford A, Easton DF, Tyrer J, Bolton KL, Harrington P, Eccles D, Chen A, Molina AN, Davila BN, Arango H, Tsai YY, Chen Z, Risch HA, McLaughlin J, Narod SA, Ziogas A, Brewster W, Gentry-Maharaj A, Menon U, Wu AH, Stram DO, Pike MC, Beesley J, Webb PM, Chen X, Ekici AB, Thiel FC, Beckmann MW, Yang H, Wentzensen N, Lissowska J, Fasching PA, Despierre E, Amant F, Vergote I, Doherty J, Hein R, Wang-Gohrke S, Lurie G, Carney ME, Thompson PJ, Runnebaum I, Hillemanns P, Dürst M, Antonenkova N, Bogdanova N, Leminen A, Butzow R, Heikkinen T, Stefansson K, Sulem P, Besenbacher S, Sellers TA, Gayther SA, Pharoah PD, Wellcome Trust Case-Control Consortium, Australian Cancer Study (Ovarian Cancer), Australian Ovarian Cancer Study Group, Ovarian Cancer Association Consortium (OCAC), Ovarian Cancer Association Consortium (OCAC) (2010). A genome-wide association study identifies susceptibility loci for ovarian cancer at 2q31 and 8q24.. Nat Genet.

[37] Sakamoto H, Yoshimura K, Saeki N, Katai H, Shimoda T, Matsuno Y, Saito D, Sugimura H, Tanioka F, Kato S, Matsukura N, Matsuda N, Nakamura T, Hyodo I, Nishina T, Yasui W, Hirose H, Hayashi M, Toshiro E, Ohnami S, Sekine A, Sato Y, Totsuka H, Ando M, Takemura R, Takahashi Y, Ohdaira M, Aoki K, Honmyo I, Chiku S, Aoyagi K, Sasaki H, Ohnami S, Yanagihara K, Yoon KA, Kook MC, Lee YS, Park SR, Kim CG, Choi IJ, Yoshida T, Nakamura Y, Hirohashi S, Study Group of Millennium Genome Project for Cancer (2008). Genetic variation in PSCA is associated with susceptibility to diffuse-type gastric cancer.. Nat Genet.

[38] Chen W, Xu W, Tao Q, Liu J, Li X, Gan X, Hu H, Lu Y (2009). Meta-analysis of the association of the *HTRA1* polymorphisms with the risk of age-related macular degeneration.. Exp Eye Res.

[39] Tang NP, Zhou B, Wang B, Yu RB (2009). *HTRA1* promoter polymorphism and risk of age-related macular degeneration: a meta-analysis.. Ann Epidemiol.

[40] Tong Y, Liao J, Zhang Y, Zhou J, Zhang H, Mao M (2010). *LOC387715*/*HTRA1* gene polymorphisms and susceptibility to age-related macular degeneration: A HuGE review and meta-analysis.. Mol Vis.

[41] Kondo N, Honda S, Kuno S, Negi A (2009). Coding variant I62V in the complement factor H gene is strongly associated with polypoidal choroidal vasculopathy.. Ophthalmology.

[42] Kondo N, Honda S, Kuno S, Negi A (2009). Role of *RDBP* and *SKIV2L* variants in the major histocompatibility complex class III region in polypoidal choroidal vasculopathy etiology.. Ophthalmology.

[43] Seddon JM, Sharma S, Adelman RA (2006). Evaluation of the clinical age-related maculopathy staging system.. Ophthalmology.

[44] Wigginton JE, Cutler DJ, Abecasis GR (2005). A note on exact tests of Hardy–Weinberg equilibrium.. Am J Hum Genet.

[45] Zhang XJ, Huang W, Yang S, Sun LD, Zhang FY, Zhu QX, Zhang FR, Zhang C, Du WH, Pu XM, Li H, Xiao FL, Wang ZX, Cui Y, Hao F, Zheng J, Yang XQ, Cheng H, He CD, Liu XM, Xu LM, Zheng HF, Zhang SM, Zhang JZ, Wang HY, Cheng YL, Ji BH, Fang QY, Li YZ, Zhou FS, Han JW, Quan C, Chen B, Liu JL, Lin D, Fan L, Zhang AP, Liu SX, Yang CJ, Wang PG, Zhou WM, Lin GS, Wu WD, Fan X, Gao M, Yang BQ, Lu WS, Zhang Z, Zhu KJ, Shen SK, Li M, Zhang XY, Cao TT, Ren W, Zhang X, He J, Tang XF, Lu S, Yang JQ, Zhang L, Wang DN, Yuan F, Yin XY, Huang HJ, Wang HF, Lin XY, Liu JJ (2009). Psoriasis genome-wide association study identifies susceptibility variants within *LCE* gene cluster at 1q21.. Nat Genet.

[46] Mantel N, Haenszel W (1959). Statistical aspects of the analysis of data from retrospective studies of disease.. J Natl Cancer Inst.

[47] DerSimonian R, Laird N (1986). Meta-analysis in clinical trials.. Control Clin Trials.

[48] Whittemore AS (1983). Estimating attributable risk from case-control studies.. Am J Epidemiol.

[49] Ioannidis JP, Patsopoulos NA, Evangelou E (2007). Heterogeneity in meta-analyses of genome-wide association investigations.. PLoS ONE.

[50] Higgins JP, Thompson SG, Deeks JJ, Altman DG (2003). Measuring inconsistency in meta-analyses.. BMJ.

[51] Sakurada Y, Kubota T, Mabuchi F, Imasawa M, Tanabe N, Iijima H (2008). Association of *LOC387715* A69S with vitreous hemorrhage in polypoidal choroidal vasculopathy.. Am J Ophthalmol.

[52] Lee KY, Vithana EN, Mathur R, Yong VH, Yeo IY, Thalamuthu A, Lee MW, Koh AH, Lim MC, How AC, Wong DW, Aung T (2008). Association analysis of *CFH, C2, BF*, and *HTRA1* gene polymorphisms in Chinese patients with polypoidal choroidal vasculopathy.. Invest Ophthalmol Vis Sci.

[53] Hardy J, Singleton A, Gwinn-Hardy K (2003). Ethnic differences and disease phenotypes.. Science.

[54] Kanda A, Chen W, Othman M, Branham KE, Brooks M, Khanna R, He S, Lyons R, Abecasis GR, Swaroop A (2007). A variant of mitochondrial protein *LOC387715*/*ARMS2*, not *HTRA1*, is strongly associated with age-related macular degeneration.. Proc Natl Acad Sci USA.

[55] Fritsche LG, Loenhardt T, Janssen A, Fisher SA, Rivera A, Keilhauer CN, Weber BH (2008). Age-related macular degeneration is associated with an unstable *ARMS2* (*LOC387715*) mRNA.. Nat Genet.

[56] Kanda A, Stambolian D, Chen W, Curcio CA, Abecasis GR, Swaroop A (2010). Age-related macular degeneration-associated variants at chromosome 10q26 do not significantly alter *ARMS2* and *HTRA1* transcript levels in the human retina.. Mol Vis.

[57] Yang Z, Tong Z, Chen Y, Zeng J, Lu F, Sun X, Zhao C, Wang K, Davey L, Chen H, London N, Muramatsu D, Salasar F, Carmona R, Kasuga D, Wang X, Bedell M, Dixie M, Zhao P, Yang R, Gibbs D, Liu X, Li Y, Li C, Li Y, Campochiaro B, Constantine R, Zack DJ, Campochiaro P, Fu Y, Li DY, Katsanis N, Zhang K (2010). Genetic and functional dissection of HTRA1 and LOC387715 in age-related macular degeneration.. PLoS Genet.

[58] Friedrich U, Myers CA, Fritsche LG, Milenkovich A, Wolf A, Corbo JC, Weber BH (2011). Risk- and non-risk-associated variants at the 10q26 AMD locus influence *ARMS2* mRNA expression but exclude pathogenic effects due to protein deficiency.. Hum Mol Genet.

